# Efficacy and safety of vacuum sealing drainage combined with skin grafting for the treatment of limb burns

**DOI:** 10.12669/pjms.41.11.12555

**Published:** 2025-11

**Authors:** Liyong Zhu, Shunyang Zheng, Yongpan Li, Pei Liu

**Affiliations:** 1 Liyong Zhu, Emergency Department (ED), Yongkang First People’s Hospital, Yongkang, Zhejiang Province 321300, P.R. China; 2 Shunyang Zheng, Emergency Department (ED), Yongkang First People’s Hospital, Yongkang, Zhejiang Province 321300, P.R. China; 3 Yongpan Li, Emergency Department (ED), Yongkang First People’s Hospital, Yongkang, Zhejiang Province 321300, P.R. China; 4 Pei Liu, Emergency Department (ED), Yongkang First People’s Hospital, Yongkang, Zhejiang Province 321300, P.R. China

**Keywords:** Burns, Limb, Skin grafting, Vacuum sealing drainage

## Abstract

**Objective:**

Exploring the efficacy and safety of vacuum sealing drainage (VSD) combined with skin grafting for limb burns.

**Methodology:**

This retrospective cohort study collected clinical records of 140 patients with limb burns who underwent skin grafting surgery in Yongkang First People’s Hospital from November, 2021 to October, 2024. Among them, 70 patients who received the first stage VSD combined with the second stage skin grafting treatment (VSD group) were matched in a 1:1 ratio with the queue of patients who received a traditional dressing change treatment combined with the second stage skin grafting treatment (conventional group). The healing time, granulation growth time, length of hospital stay, scar hyperplasia (as assessed by the Vancouver Scar Scale, VSS), degree of pain and incidence of complications were compared.

**Results:**

The healing time, granulation growth time, length of hospital stay and VSS score of the VSD group were lower than those of the conventional group (P<0.05). The VAS scores of the VSD group were lower compared to the conventional group at one, three and seven days after treatment (P<0.05). VSD combined with skin grafting was associated with a significantly lower (18.6%) incidence of complications than the conventional treatment (34.3%) (P<0.05).

**Conclusions:**

VSD combined with skin grafting is safer than the traditional treatment approach and is more efficient in shortening the wound healing process and reducing pain and scar formation in patients with limb burns.

## INTRODUCTION

Limb burns are associated with high disability and mortality rates.^1^,^2^ Without timely and effective intervention, they may lead to wound infection and affect the outcome.^1^-^3^ Therefore, early implementation of safe and effective treatment for patients with limb burns is crucial.

Skin grafting is one of the primary measures for treating burns, as it can effectively remove inactive necrotic tissue, seal open wounds, promote early wound healing and alleviate stress damage.^4^-^6^ However, postoperative pressure bandaging and routine dressing changes can easily cause wound infection and increased pain, negatively impacting the postoperative wound recovery and reducing the survival rate of skin grafts.^7^,^8^ Vacuum sealing drainage (VSD) uses negative pressure drainage dressings to cover the wound, creating a tight sealing effect and preventing wound infection, while the continuous cleaning of wound exudate during VSD accelerates the growth of granulation tissue in the wound, thereby ensuring the rehabilitation effect.^8^,^9^

At present, there is insufficient evidence on the effectiveness and safety of VSD combined with skin grafting for treating limb burns. This study retrospectively analyzed the clinical data of patients with limb burns who underwent VSD combined with skin grafting surgery to clarify the therapeutic efficacy and safety of this treatment modality.

## METHODOLOGY

This retrospective cohort study included data from patients who underwent skin grafting at Yongkang First People’s Hospital from November, 2021 to October, 2024. All cases were consecutively selected from electronic medical records of patients with limb burns treated at our institution between November, 2021 and October, 2024. Inclusion and exclusion criteria were applied in chronological order to ensure consistency. Data collection was standardized, with all clinical information recorded by trained medical staff following a uniform protocol to minimize variability. Although blinding of clinicians during treatment allocation was not possible due to the retrospective nature of the study, objective outcome measures and consistent data collection procedures were implemented to reduce the risk of information bias.

### Ethical Approval:

The ethics committee of our hospital approved the study with number EC2025-(LW)002-01(K) on January 18^th^, 2025.

According to the treatment records, the patients were divided into the VSD group (first-stage VSD combined with second-stage skin grafting treatment) and the conventional group (traditional dressing change treatment combined with second-stage skin grafting treatment). Two groups were matched in a 1:1 ratio based on age, gender, burn type, and burn area as the primary matching criteria. These variables were selected because they were consistently available in the medical records and are clinically relevant to burn prognosis. Other potentially important prognostic factors, such as precise burn depth grading, presence of inhalation injury, initial hemodynamic status, and baseline nutritional status, were not included due to incomplete data in some cases, which could have introduced additional bias if applied inconsistently.

### Inclusion criteria:

- Patients with limb burns.
- Burn depth of II-III degrees.
- Follow-up for more than six months and complete clinical data.

### Exclusion criteria:

- Patients with organic lesions in organs such as the heart, kidneys and liver.
- Patients with peripheral vascular diseases, diabetes and other diseases that affect wound healing.
- Patients with hematological and immune system disorders.
- Breastfeeding and pregnant women.

### Traditional treatment:

The first stage of traditional dressing change therapy was combined with the second stage of skin transplantation treatment. After routine anesthesia, two hundred thousand units of adrenaline saline swelling solution was injected into the subcutaneous area of the wound. The wound and surrounding area were disinfected with iodine three times before surgery. A roller knife was used to clean the degenerated dermis of the wound until viable subcutaneous tissue appeared, followed by electrocoagulation for hemostasis treatment. The wound was covered with 1:10000 adrenaline solution gauze and washed with antibiotic saline, physiological saline and wound flushing solution to remove foreign objects, necrotic and infected inactivated tissues. When the blood supply to the patient’s distal limbs was affected, incision and decompression were performed to avoid ischemic necrosis of the limbs. The wound was cleaned and the dressing was changed every day. Skin grafting was done on fresh granulation tissue from the burn site. The wound was excised to normal tissue using the electrosurgical knife and covered with a thick to medium-thickness skin patch. Multiple layers of dressings were wrapped around the injured area.

### First-stage VSD plus second-stage skin transplantation:

The routine debridement and wound hemostasis were performed. Symptomatic treatment included infusion, anti-shock and anti-infection measures as needed. After routine anesthesia, the burn wound was cleaned. After removing necrotic and inactivated tissue, complete hemostasis was achieved through electrocoagulation. The size of the wound was measured using sterile gauze and the negative pressure dressing was cut to ensure that it is 2 cm larger than the normal skin around the wound and wound edge. A skin-binding machine was used to apply the dressing in a tension-free state around the wound and properly fix it with a semi-permeable adhesive film. The suction cup of the drainage tube was connected at a low position and the drainage tube was attached to the NPWT host (Nanjing Ningxing Medical Technology Co., Ltd., China). The pressure was adjusted to 50 mmHg and the negative pressure value was adjusted to 75-125 mmHg based on the drainage location and wound condition. The collapse of negative pressure material indicated that it was suitable for smooth drainage. After VSD, continuous or intermittent irrigation was performed according to the condition of the wound. After continuously treatment for 5-7 days, the negative pressure device was removed and the necrotic tissue was thoroughly cleaned and skin transplantation was performed. If there was no fresh granulation tissue growth after seven days, the VSD treatment was continued until the tissue was healthy.

### Postoperative wound repair:

Autologous skin pieces (thickness 0.25 mm) were cut using MEEK Micro - Skin Patch (Humeca, Netherlands), placed on a disinfection plate, soaked in physiological saline for 0.5-1 hour and cut to the appropriate size. The specialized adhesive was evenly sprayed onto the surface of the microfiber and stuck onto the polyamide double crepe gauze. After 5-10 minutes, the micro skin completely expands by forming equal distances between the skin patches. The micro skin was soaked in physiological saline, transplanted directly onto the surface of the wound and the wound was wrapped with a sterile dressing. All second-stage skin grafting procedures were performed by the same team of experienced surgeons to ensure uniformity of surgical technique. Donor sites were standardized across both groups, with split-thickness grafts harvested from common anatomical regions such as the thigh or abdomen, depending on the patient’s clinical condition. The expansion ratio of grafts was consistently maintained at 1:1.5 to 1:2 to provide comparable wound coverage. Postoperative dressing and wound care protocols were identical in both groups, including the use of the same dressing materials and a standardized schedule for dressing changes, following institutional clinical practice.

Collected indices included the patient’s wound healing time, granulation tissue growth time, length of hospital stay, scar hyperplasia, pain level and incidence of complications. For scar hyperplasia, the Vancouver Scar Scale (VSS) was used for evaluation at six months after surgery, with a score range of 0-15 points (the lower the score, the milder the scar hyperplasia). For the degree of pain, the visual analog scale (VAS) was used to evaluate the pain level at one, three and seven days after treatment, with a score range of 0-10 points. A lower score indicated milder pain. Complications were defined and assessed using explicit criteria. Infection was diagnosed when clinical signs such as increased redness, warmth, and purulent discharge were present, requiring systemic antibiotic treatment, and was confirmed by the attending physician. Exudation was considered significant if wound drainage exceeded 100 mL per day for three consecutive days. Pruritus was assessed using a visual analog scale (VAS) for itch intensity, with scores ≥3 indicating clinically relevant pruritus. Complications were evaluated at three key time points: immediately after VSD therapy, at the time of skin grafting, and during follow-up at 1, 3, and 7 days post-surgery. To ensure inter-observer reliability, all data were collected by trained medical staff following standardized protocols, and discrepancies were resolved in consultation with a senior physician.

### Statistical Analysis:

SPSS 25.0 software program (IBM Corp., Armonk, NY, USA) was used to perform statistical analysis on the collected data. The Shapiro-Wilk test was used to evaluate the normality of quantitative data. Data that conformed to a normal distribution were represented by mean ± standard deviation and intergroup comparisons were conducted using an independent sample t-test; Data that did not conform to a normal distribution were represented by median and interquartile range and the Mann-Whitney U test was used for intergroup comparison. Chi-square test or Fisher’s exact test was used to evaluate the differences in covariate distribution between groups. A two-sided p-value of<0.05 was considered statistically significant.

## RESULTS

This study included 140 patients aged 18-89, with an average age of 48.6 ± 14.6 years. Based on the received treatment, patients were divided into the VSD and conventional groups in a 1:1 ratio, with 70 patients in each group. There was no statistically significant difference between the two groups in terms of basic characteristics such as age, gender, burn to hospital admission time, burn type and burn area proportion (P>0.05) (Table-I).

**Table-I:** Comparison of basic characteristics between two groups.

Characteristics	VSD group (n=70)	Conventional group (n=70)	t/χ^2^/Z	P
Age (years), mean±SD	47.6±14.3	49.6±14.9	-0.815	0.416
Gender, n(%)			1.120	0.290
Male	48 (68.6)	42 (60.0)		
Female	22 (31.4)	28 (40.0)		
Burn to hospital admission time (hours), M(IQR)	7 (5-9)	7.5 (5-9)	-0.573	0.566
Cause of injury, n(%)			1.808	0.613
Thermal Burn	47 (67.1)	51 (72.9)		
Chemical Burn	4 (5.7)	5 (7.1)		
Electrical Burn	18 (25.7)	12 (17.1)		
Radiation Burn	1 (1.5)	2 (2.9)		
Percentage of burnt area (%), M(IQ)	6 (3-12)	8 (5-15)	-1.776	0.076

VSD: vacuum sealing drainage

**Fig.1:**
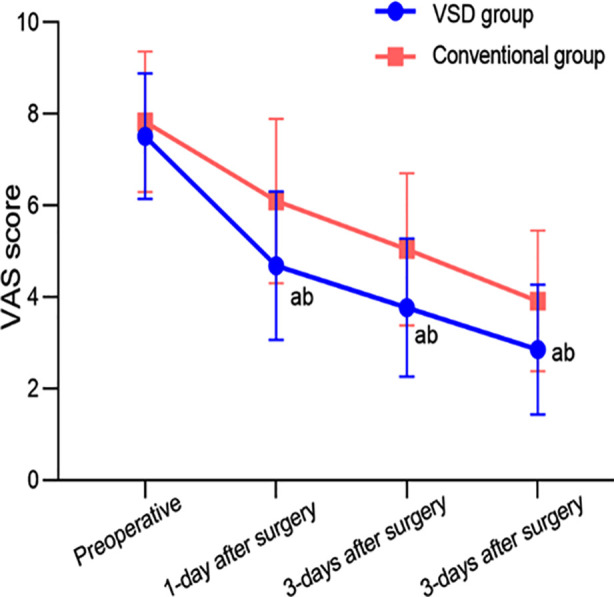
Comparison of pain levels between the two groups.

The VSD group had lower VSS scores, wound healing time, granulation growth time and the length of hospital stay compared to the conventional group (P<0.05) (Table-II). The VAS scores at day one, day three and day seven after surgery in both groups were lower than before treatment and considerably lower in the VSD group compared to the conventional group (P<0.05) (Fig.1).

**Table-II:** Wound healing process and hospitalization times

Variables	VSD group (n=70)	Conventional group (n=70)	Z/t	P
Time required for wound healing (days), M(IQR)	19 (13-25)	25 (14-30)	-2.153	0.031
Granulation growth time (days), M(IQR)	18 (12-24)	24 (13-29)	-2.249	0.025
Duration of hospitalization (days), mean±SD	28.6±8.6	33.2±9.1	-3.100	0.002
VSS (scores), M(IQR)	4 (3-5)	5 (4-6)	-4.100	<0.001

VSD: vacuum sealing drainage; VSS: Vancouver Scar Scale; SD: standard deviation; M(IQR): median of the interquartile range (IQR).

Compared with before treatment in the same group, ^a^*P*<0.05; Compared to conventional groups, ^b^*P*<0.05. VAS: visual analog scale; VSD: vacuum sealing drainage.

As shown in Table-III, both groups had comparable incidences of specific complications, such as increased exudation, edema, pruritus and infections (P>0.05). However, VSD combined with skin grafting was associated with a significantly lower total rate of complications compared to the conventional treatment approach (18.6% vs 34.3%, respectively) (P<0.05).

**Table-III:** Complication rates.

Complication	VSD group (n=70)	conventional group (n=70)	χ^2^	P
Increased exudation	1 (1.4)	3 (4.3)	0.257	0.612**
Pruritus	2 (2.9)	4 (5.7)	0.174	0.676**
Edema	2 (2.9)	4 (5.7)	0.174	0.676**
Infection	8 (11.4)	13 (18.6)	1.401	0.237*
Total number of occurrences	13 (18.6)	24 (34.3)	4.445	0.035*

* Pearson’s Chi-square test;

** Fisher’s Exact Test. VSD: vacuum sealing drainage

## DISCUSSION

This study showed that in patients with limb burns, the first-stage VSD combined with second-stage skin grafting was more efficient in improving wound healing time, granulation growth time and was associated with lower degree of scar hyperplasia and shorter hospital stay compared to the traditional dressing change treatment combined with the second-stage skin grafting treatment. Additionally, patients in the VSD group reported more effective postoperative pain relief and a lower incidence of complications. These results are consistent with the previous research results by Jiao et al.^10^ and Sun et al.^11^ and further confirm that combining first-stage VSD and second-stage skin grafting can reduce complications, especially wound infections, promote smooth growth of granulation tissue and provide favorable conditions for second-stage skin grafting.

However, the study by Tapking et al.^12^ reported that VSD did not have significant advantages in wound healing time, pain and scar formation compared to conventional treatment. This discrepancy may be attributed not only to differences in sample size and burn severity, but also to methodological variations such as patient selection criteria and burn depth categorization. In addition, the influence of different nursing models, including the consistency and intensity of postoperative wound care, could substantially affect outcomes. Another important factor is patient adherence to VSD therapy, which can vary depending on tolerance of negative pressure, noise, or mobility restrictions. These aspects may partly explain the divergent results observed across studies. Furthermore, mechanistically, the combination of VSD and skin grafting may accelerate granulation tissue formation and wound healing through enhanced local blood circulation, improved tissue oxygenation, and reduction of bacterial load in the wound environment.^10^,^11^ It is plausible that VSD technology can effectively remove wound exudate and necrotic tissue by creating a closed negative pressure environment, reducing bacterial growth.^13^ At the same time, continuous negative pressure can promote local blood circulation, stimulate granulation tissue to accelerate growth and create favorable wound conditions for subsequent skin grafting.^11^,^13^

This study compared the degree of scar hyperplasia between two groups of patients after six months of treatment. The record shows that the degree of scar hyperplasia in the VSD group is lower than in the conventional group. Currently, the main approach to prevent wound infection is through debridement and daily dressing changes.^7^,^14^ However, treating tissues and exudate from deep burns presents certain challenges.^14^,^15^ As a novel healing and drainage technique, VSD can remove exudate and necrotic tissue in a sealed environment.^11^,^13^,^16^ Studies showed that VSD combined with skin grafting can maintain skin tension and elasticity by timely repairing the wound and reducing mechanical stimulation caused by wound contraction, thus reducing the risk of scar contraction and hyperplasia.^13^,^16^

The VAS scores of the patients in the VSD group were lower than those of the conventional group at day one, day three and day seven after surgery. This result is consistent with previous observations that negative pressure suction of VSD can promote exudate discharge, reduce tissue edema, lower tissue tension and alleviate pain caused by compression.^16^,^17^ Moreover, under the coverage of VSD dressing, the wound is in a relatively stable pressure environment, which also helps to alleviate pain.^17^

The main complication reported in this study was infection (21 cases), followed by edema and skin itching (6 cases each). The incidence of complications in the VSD group was significantly lower than in the conventional group, which can be attributed to the reduced risk of infection associated with the VSD technology: negative pressure suction of VSD allows for keeping the wound clean and minimizing the opportunity for bacterial growth.^16^ Consistent with this study, Zhou Q et al.^18^ showed that VSD promotes wound healing and reduces other complications caused by long-term nonhealing of the wound. Together, these results indicate that VSD combined with skin grafting therapy is highly safe and can reduce patient risks during the treatment process.

Since VSD technology requires special negative pressure drainage devices, closed dressings and other materials, the treatment cost is relatively high and may be associated with a certain economic burden on the patients.^16^-^20^ In addition, the operation process of VSD is cumbersome and requires a high level of technical qualifications of medical staff. Improper operation may result in poor sealing of the VSD device and blockage of the drainage tube, or improper aseptic techniques during the operation process, which may lead to bacterial growth and infection.^18^-^21^ However, since this study clearly indicates the superiority of the VSD combined with skin grafting, clinical doctors can prioritize using the first-stage VSD combined with the second-stage skin graft treatment method when treating patients with limb burns. Nevertheless, the interpretation of these findings requires caution. Because patients with common comorbidities such as diabetes and peripheral vascular disease were excluded, the present results are primarily applicable to populations with relatively low comorbidity, and their extrapolation to high-risk groups should be undertaken with care. In addition, different burn mechanisms—including thermal, chemical, and electrical injuries—may exert distinct biological effects on granulation tissue formation and infection susceptibility, yet stratified analysis by burn type was not feasible due to the limited subgroup sizes in this study. Furthermore, functional outcomes such as joint mobility, hand function, and quality-of-life indicators (e.g., SF-36 scores) were not assessed, although these are particularly important for evaluating long-term recovery in limb burn patients. Finally, although a negative pressure range of 75–125 mmHg was applied according to clinical practice, direct comparisons across different pressure levels were not performed owing to the sample size; future studies should therefore investigate whether pressure-specific effects exist to optimize treatment parameters. Collectively, these limitations underscore the need for larger, multi-center studies with longer follow-up and broader inclusion criteria to provide a more comprehensive understanding of the long-term efficacy and safety of VSD therapy combined with skin grafting.

### Strengths of this study:

The strengths of this study include the use of a matched cohort design to minimize baseline differences, the inclusion of a relatively large sample size of 140 patients, and the focus on clinically relevant short-term outcomes such as wound healing, pain reduction, and complication rates, which are critical for guiding immediate clinical practice. Nevertheless, further research is warranted in several areas. Longer follow-up studies are needed to assess long-term functional recovery, quality of life, and complications in burn patients treated with VSD therapy. Future investigations should also evaluate the effectiveness of VSD in high-risk populations with comorbidities, explore the impact of different negative pressure levels on outcomes, and incorporate more detailed stratification by burn type and additional prognostic variables such as burn depth, inhalation injury, and baseline health status.

### Limitations

Firstly, as a single-center retrospective study, it is subject to inherent risks of selection and information bias despite the use of a matched cohort design. Further validation through prospective, multi-center studies is required. Secondly, some potentially important prognostic factors—such as precise burn depth grading, inhalation injury, baseline hemodynamic status, and nutritional status—were not incorporated into the matching process due to incomplete data availability, which may have introduced residual confounding. Thirdly, the external validity of the findings is limited by factors such as geographical location and hospital care level, which may affect generalizability to other populations and institutions. Moreover, although this study focused on short-term clinical outcomes, long-term follow-up data, including functional recovery (e.g., joint range of motion and limb function) and quality of life indicators, were not collected. These outcomes are critical for comprehensively evaluating the long-term benefits of VSD combined with skin grafting and should be included in future research. Additionally, patient-reported outcomes, such as adherence, satisfaction, and subjective experiences with VSD therapy, were not assessed, despite their importance in understanding the broader impact of treatment. Finally, recurrence, reinfection, and the need for re-intervention after VSD removal were not monitored during follow-up. These outcomes are clinically relevant to assessing the long-term safety and efficacy of VSD therapy and should be systematically incorporated into future studies.

## CONCLUSION

An association was observed under the conditions of this study, where first-stage VSD combined with second-stage skin grafting was associated with improved wound healing, reduced pain and scar hyperplasia, and fewer complications compared to the conventional treatment approach. Further high-quality randomized controlled trials with larger sample sizes and extended follow-up times are needed to validate these findings.

### Authors’ contributions:

**LZ:** Literature search, Study design and manuscript writing.

**SZ, PL and YL:** Data collection, data analysis and interpretation. Critical Review.

**LZ:** was involved in the manuscript revision and validation and is responsible for the integrity of the study.

All authors have read and approved the final manuscript.

## References

[1] Shen YM, Qin FJ, Du WL, Wang C, Zhang C, Chen H (2019). Limb salvage strategies for patients with high voltage electric burns of extremities on the verge of amputation. Zhonghua Shao Shang Za Zhi..

[2] Murtaugh B, Warthman R, Boulter T (2023). Rehabilitation Management of the Burned Hand. Phys Med Rehabil Clin N Am..

[3] Ahmed M, Zahid I (2023). Responding to burns - is our practice adequate?. J Pak Med Assoc.

[4] Schlottmann F, Bucan V, Vogt PM, Krezdorn N (2021). A Short History of Skin Grafting in Burns:From the Gold Standard of Autologous Skin Grafting to the Possibilities of Allogeneic Skin Grafting with Immunomodulatory Approaches. Medicina (Kaunas)..

[5] Dogan S, Sjoberg F, El-Serafi AT, Sjöberg Z, Abdelrahman I, Steinvall I (2024). Advancements in skin grafting:Development and application of a novel two-blade dermatome for concurrent split-thickness and dermal graft harvesting. Burns..

[6] Ozhathil DK, Tay MW, Wolf SE, Branski LK (2021). A Narrative Review of the History of Skin Grafting in Burn Care. Medicina (Kaunas)..

[7] Orbay H, Corcos AC, Ziembicki JA, Egro FM (2024). Challenges in the Management of Large Burns. Clin Plast Surg..

[8] Nguyen TQ, Franczyk M, Lee JC, Greives MR, O'Connor A, Gottlieb LJ (2015). Prospective randomized controlled trial comparing two methods of securing skin grafts using negative pressure wound therapy:vacuum-assisted closure and gauze suction. J Burn Care Res.

[9] Gómez-Ortega V, Vergara-Rodriguez MJ, Mendoza B, García T (2021). Effect of Negative Pressure Wound Therapy in Electrical Burns. Plast Reconstr Surg Glob Open..

[10] Jiao H, Tian T, Chen C, Ji G (2022). Effect of vacuum sealing drainage plus skin grafting on deep burns and analysis of risk factors for postoperative infection. Am J Transl Res.

[11] Sun T, Ying W, Wang S, Chen C, Sun P, Tan J (2023). Clinical Application of Vacuum Sealing Drainage for the Treatment of Deep Burn Wounds. Am Surg..

[12] Tapking C, Endlein J, Warszawski J, Kotsougiani-Fischer D, Gazyakan E, Hundeshagen G (2024). Negative pressure wound therapy in burns:a prospective, randomized-controlled trial. Burns..

[13] Nuhiji E (2024). Trends and Innovation in Negative Pressure Wound Therapy:A Review of Burn Wound Management. Adv Wound Care (New Rochelle)..

[14] Ding X, Cui H, Ma P, Chen X, Sun Y, Qu M (2022). Efficacy of dexmedetomidine versus midazolam when combined with butorphanol for sedation and analgesia during burn dressing changes:A randomized clinical trial. Front Pharmacol..

[15] Palmieri TL (2023). Acute care for burn patients:fluids, surgery and what else?. Curr Opin Crit Care.

[16] Guo Y, Liu X, Chen L (2023). Treating Hand High-voltage Electrical Burn by Combination of Radial Artery Perforator Flap, Artificial Dermis and Vacuum Sealing Drainage. Plast Reconstr Surg Glob Open..

[17] Xu J, Hua Y, Lei J, Peng X, Cheng L, Jiang Q (2023). Effects of skin flap grafting combined with vacuum sealing drainage on ulcer area, pain level and serum inflammation in diabetic foot patients. Am J Transl Res.

[18] Zhou Q, Li SS, Wang Q, Lu Y, Si YN, Wang LN (2019). Influence of cluster nursing intervention on inadequate drainage in vacuum sealing drainage for inpatients in burn unit. Zhonghua Shao Shang Za Zhi..

[19] Liu MD, Yang XK, Han F, Fang ZQ, Zhang Y, Hu DH (2018). Strategy for wound repair of skin and soft tissue defect and systematic rehabilitation treatment for functional reconstruction of patients with severe burn or trauma on knees. Zhonghua Shao Shang Za Zhi..

[20] Chen S, Zheng LW, Liu W, Chen ZH (2019). Clinical effects of artificial dermis combined with vacuum sealing drainage and autologous split-thickness skin graft in repair of scar contracture deformity after extensive burn. Zhonghua Shao Shang Za Zhi..

[21] Lou J, Zhu X, Xiang Z, Fan Y, Song J, Huang N (2024). The efficacy and safety of negative pressure wound therapy in paediatric burns:a systematic review and meta-analysis of randomized controlled trials. BMC Pediatr..

